# Micronutrient and Inflammation Status Following One Year of Complementary Food Supplementation in 18-Month-Old Rural Bangladeshi Children: A Randomized Controlled Trial †

**DOI:** 10.3390/nu12051452

**Published:** 2020-05-18

**Authors:** Rebecca K. Campbell, Saijuddin Shaikh, Kerry Schulze, Margia Arguello, Hasmot Ali, Lee Wu, Keith P. West, Parul Christian

**Affiliations:** 1 Department of International Health, Johns Hopkins Bloomberg School of Public Health, 615 N. Wolfe St, Baltimore, MD 21205, USA; rebecca.campbell@mssm.edu (R.K.C.); kschulz1@jhu.edu (K.S.); marguello@jhu.edu (M.A.); lwu2@jhu.edu (L.W.); kwest1@jhu.edu (K.P.W.J.); 2 JiVitA Project, Gaibandha-5700, Bangladesh; saiju.jivita@gmail.com (S.S.); hasmot.jivita@gmail.com (H.A.)

**Keywords:** complementary foods, supplementation, micronutrients, inflammation, children, growth

## Abstract

**Background:** Four fortified complementary food supplements (CFSs) in a randomized controlled trial (RCT) were found to improve childhood linear growth in rural Bangladesh. We hypothesized children receiving these supplements would have improved micronutrient status. **Methods:** In the RCT, we assessed hemoglobin and serum ferritin, retinol, zinc, C-reactive protein (CRP), and α-1-acid glycoprotein (AGP) at endline (18 mo) in a subsample of children (*n* = 752). The impact of supplementation on mean concentrations and the prevalence of nutrient deficiency and inflammation were evaluated using adjusted generalized estimating equation (GEE) linear and log-binomial regression models. **Results:** In the control arm at age 18 months, 13% of children were anemic (hemoglobin < 110 g/L), and 6% were iron (inflammation-adjusted ferritin < 12 μg/L), 8% vitamin A (inflammation-adjusted retinol < 0.70 μmol/L), and 5% zinc (zinc < 9.9 μmol/L) deficient. The prevalence of inflammation by CRP (>5 mg/L) and AGP (>1 g/L) was 23% and 66%, respectively, in the control group. AGP trended lower in CFS groups (*p* = 0.04), while CRP did not. Mean ferritin (*p* < 0.001) and retinol (*p* = 0.007) were higher in all supplemented groups relative to control, whereas hemoglobin improved with two of the four CFSs (*p* = 0.001), and zinc was equal or lower in supplemented groups relative to control (*p* = 0.017). **Conclusions:** CFSs improved iron status and vitamin A concentrations and lowered inflammation in a context of low underlying nutrient deficiency but high inflammation.

## 1. Introduction

Micronutrient deficiencies, especially vitamin A, iron, zinc, and iodine, commonly affect children in low- and middle-income countries, often coexisting with stunting and wasting [1,2]. Worldwide, 33% and 17% of children < 5 y have vitamin A and zinc deficiency, respectively, and 18% have anemia [1], with serious consequences for health and development. Iron deficiency impairs cognitive development and immune function [3,4,5], vitamin A is required for immune function [6,7], and mild zinc deficiency is associated with stunted linear growth, impaired immune function, and increased severity of diarrhea and pneumonia [8,9]. Prevalence of these deficiencies in South Asia are among the highest worldwide [2,10,11].

Complementary food supplements (CFSs) may prevent undernutrition during the 6–24 mo period when diverse foods and frequent meals are needed along with breastmilk to meet nutritional requirements. CFSs contain supplemental energy and protein and are fortified with multiple micronutrients [12]. Field trials of CFSs have reported positive impacts on linear growth and prevention of stunting and wasting [13,14,15,16,17]. A few studies, all in Africa, have examined impacts on micronutrient status. For example, in Chad, adding a CFS to a standard family food package in a setting of food insecurity improved young children’s hemoglobin (Hb) and reduced anemia relative to those receiving the food package alone [18]. In Malawi, 12 weeks of supplementation with lipid-based fortified spreads improved Hb [19], and in Ghana, children receiving a CFS had improved iron but no change in zinc status [20].

We conducted a randomized controlled trial in 2012–2014 in Bangladesh to test the impact of four CFS formulations on linear growth and stunting [21]. Supplementation significantly improved linear growth with a 5–6 percentage point reduction in stunting prevalence at 18 mo of age in two CFS groups relative to control [21]. In a subsample of children, we assessed serum micronutrient status and inflammation at 18 mo to evaluate the impact on these of one year of CFS supplementation. 

## 2. Methods

***Setting*** Four CFSs were tested in an unblinded, community-based, cluster-randomized controlled trial in the Gaibandha and Rangpur districts of rural northwest Bangladesh [21]. Children were enrolled between 2 September 2012, and 15 May 2013, and assigned to one of five study arms by their sector of residence (*n* = 596 sectors). All children living in the study area, reaching age 6 mo during the enrollment period, were eligible. Mothers in all arms received child feeding counseling (CFC); children in four of five arms additionally received a daily CFS. The CFSs were equivalent in energy and micronutrients (Supplementary Table S1), providing approximately 75% of the daily requirement [22], but differed in main ingredients and source: (1) chickpea-based (CP) and (2) rice- and lentil-based (RL), developed and produced in Bangladesh; (3) peanut-based Plumpy’doz (PD) manufactured by Nutriset (Malaunay, France); (4) fortified wheat-soy blend (WSB++) from the World Food Programme. The primary study outcomes were length and stunting prevalence at 18 mo following one year of supplementation [21].

In a 93-sector area (the “substudy”)—selected to yield a target sample size of 750 participants (150 per arm), to be contiguous and accessible by road, socioeconomically representative, and balanced by intervention arm—additional assessments were conducted for secondary outcomes, including blood collection at 18 mo of age to assess biochemical markers of nutritional status. This sample size of 750 was sufficient to show a difference of 0.21 SD or more between the intervention and control group across all biomarkers at alpha = 0.05 and beta = 0.20. No biospecimens were collected at enrollment at age 6 mo due to logistic considerations and because blood collection at such a young age is sensitive in this rural community and could lead to high refusals in study participation. All enrolled children living within the designated area were eligible for the substudy.

***Field Methods*** Children were brought to a center at 18 mo of age, and trained phlebotomists collected venous blood using trace-element-free materials. Blood was allowed to clot and transported in a cold box to the project laboratory, then centrifuged, and the serum was extracted and stored in liquid nitrogen pending shipment to Johns Hopkins. Hb was tested using a portable photometer (HemoCue, Model: Hb 301) that was factory calibrated against the International Council for Standardization in Haematology (ICSH) reference method at the time of blood draw, and a report of the output was given to mothers. According to the protocol, children with severe anemia (Hb < 70 g/L) would have been treated, but none met this criterion.

Household assets, food insecurity, water, and sanitation infrastructure, including drinking water iron content and parental education, were assessed using questionnaire-based interviews at trial enrollment in the participants’ homes. Food insecurity was measured using a validated nine-item screener about the household’s experience in the prior six months [23]. The iron content of drinking water was assessed subjectively: respondents were asked whether the household’s usual drinking water source contained “none”, “a little”, “a medium amount”, or “a lot” of iron [24]. Child anthropometry was measured at enrollment and every three months to age 18 mo.

***Laboratory Methods*** Serum was analyzed for retinol (vitamin A), ferritin (iron), zinc, C-reactive protein (CRP), and α-1 acid glycoprotein (AGP) (inflammation) at the Johns Hopkins Center for Human Nutrition micronutrient lab (Baltimore, MD, USA) using previously-described methods (Table 1) [25].

***Statistical Analysis*** Length-for-age (LAZ), weight-for-age (WAZ), and weight-for-length (WLZ) Z-score < −2 relative to the WHO growth reference standards [26] defined stunting, underweight, and wasting, respectively. Food insecurity was dichotomized into none/mild/moderate vs. severe. The highest two levels of drinking water iron (“a lot” and “a medium amount”) were combined into one category. Maternal education was dichotomized as any or none.

Initial analyses determined the extent to which the micronutrient status assessment sample was representative of the main study and balanced by randomly-assigned supplementation groups. To account for clustering within randomization clusters, generalized estimating equation (GEE) linear and logistic regression models estimated group-wise differences in baseline characteristics with clustering within sectors. Ordinal logistic regression using “*vce (cluster …)*” was used for one dependent variable with more than two levels.

Biologically implausible outliers—retinol > 3 μmol/L (*n* = 1, 0.1%) and zinc > 30.6 μmol/L (*n* = 15, 2%)—were recoded to missing. Differences in micronutrient status with continuous inflammation markers and by inflammation status (elevated CRP > 5 mg/L or elevated AGP > 1 g/L) [27] were evaluated using GEE logistic regression models. Subsequently, ferritin and retinol were adjusted according to the Biomarkers Reflecting Inflammation and Nutrition Determinants of Anemia (BRINDA) Project internal regression correction method [28,29]. Briefly, regression models were developed for ferritin (log-transformed) and retinol as functions of log-CRP and log-AGP. The absence of excess multicollinearity between CRP and AGP was confirmed with variance inflation factors (VIF). Ferritin and retinol values for children with CRP or AGP greater than the 10th percentile within the sample were replaced with corrected values. According to BRINDA guidelines, adjusting zinc for inflammation is advised only when associations between zinc and CRP or AGP are apparent [30], which they were not in the present data. Dichotomous micronutrient deficiency variables were generated, using unadjusted and inflammation-adjusted values for ferritin and retinol: ferritin < 12 μg/L [31]; Hb < 110 g/L [31]; retinol < 0.70 μmol/L [32]; zinc < 9.9 μmol/L [33]. Continuous ferritin, CRP, and AGP were log-transformed. Elevated inflammation markers were defined as CRP > 5 mg/L and AGP > 1 g/L [27]. Dichotomous inflammation (inflammation/no inflammation) was defined as elevated CRP or AGP.

Linear and log-binomial GEE models were developed to evaluate intervention effects on micronutrient and inflammation marker concentrations and status, respectively, accounting for cluster randomization. Design effects were small to moderate and provided in Supplementary Table S2. Supplementation effects as mean differences and prevalence ratios were determined by CFS type. Models were adjusted for child sex and baseline stunting. Models for ferritin, iron deficiency, Hb, and anemia were adjusted for drinking water iron. Analyses were done by intention to treat in Stata 14.1 (StataCorp, College Station, TX, USA).

Written parental consent was required for the main trial and for this sub-study. Protocols were approved by the Institutional Review Board of Johns Hopkins Bloomberg School of Public Health (Baltimore, MD, USA) and the Ethical Review Committee of icddr,b (Dhaka, Bangladesh) and were in accordance with the Helsinki Declaration.

## 3. Results

The substudy included 828 children, of whom 48.8% were female (Table 2). On enrollment at age 6 months, 27.2% were stunted, 5.2% wasted, and 20.4% underweight. Most (71.3%) came from households that owned land and had mothers with some education (76.7%), but 11.7% of households reported severe food insecurity. Supplementation adherence was high (median 90–93% in the four supplemented arms) [21]. This sample did not differ from the main trial participants on child or household characteristics (Supplementary Table S3). Allocation groups differed only on stunting prevalence (*p* = 0.007) (Table 2), which was also observed in the main trial at baseline [21].

**Figure 1:**
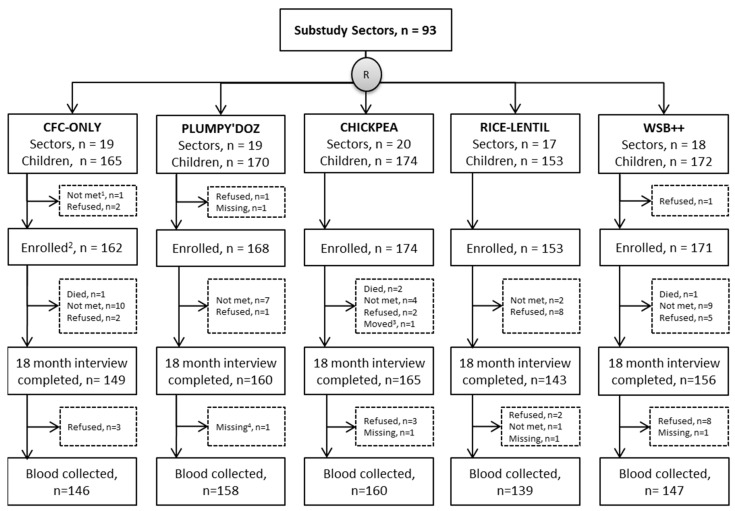
Flow diagram of participants with serum collected at 18 months of age for micronutrient status assessment in a complementary food supplementation trial in Bangladesh.

Serum samples for 752 children were collected between August 2013 and May 2014 at the end of one year of supplementation when participating children were 18 months old (Figure 1). Most missing samples were due to parental refusal (*n* = 34) or the child being unavailable within the eligibility period (*n* = 30); the remaining (*n* = 12) were due to other reasons, such as moving out of the area.

CRP and AGP were commonly elevated: 21.4% and 57.1%, respectively (Table 3). Of 60% of children with any inflammation, 3% had elevated CRP only, 39% elevated AGP only, and 18% both elevated CRP and elevated AGP. Supplementation was associated with a tendency towards lower CRP and AGP, which reached borderline-significance for CRP (*p* = 0.088). In log-binomial models, the risk of inflammation (elevated CRP or AGP) was reduced by 13–28% (*p* = 0.12) in the supplemented groups relative to control.

Inflammation was associated with higher geometric mean ferritin (40.6 vs. 31.8 μg/L), lower mean retinol (1.2 vs. 1.3 μmol/L), and lower geometric mean zinc values (12.9 vs. 13.4 μmol/L) (Supplementary Figure S1). Associations between continuous ferritin and retinol and continuous CRP and AGP were also observed (not shown). Subsequent treatment effect analyses adjusted ferritin and retinol for inflammation [28,29].

In all arms combined, 6.3% of children were iron deficient after adjusting for inflammation (6.7% unadjusted for inflammation, Supplementary Table S4), and 13.2% were anemic (Table 4). Most households (58.6%) reported detectable iron in their main source of drinking water (Table 2), and in an ancillary analysis, we found that children’s ferritin and Hb increased with increasing level of reported groundwater iron (Supplementary Figure S2), with corresponding reductions in the prevalence of iron deficiency and anemia, so perceived iron content of the household water source was included in models of supplementation effects on iron status and anemia (Table 4). Inflammation-adjusted ferritin was greater by 5.3–14.2 μg/L in the supplemented groups relative to control (*p* < 0.001) in adjusted models, with the largest effects in the Plumpy’doz and rice-lentil groups. Risk of iron deficiency (inflammation-adjusted) was reduced in all groups relative to control, with effect sizes ranging from a 27% reduction (prevalence ratio (PR): 0.73, 95% CI: 0.37–1.43) in the WSB++ group to 78% reduction (PR: 0.22, 95% CI: 0.08–0.58) in the Plumpy’doz group. Hb was higher in the supplemented groups relative to control (*p* = 0.001), with the largest differences in the Plumpy’doz and WSB++ groups. The risk of anemia was also lower in supplemented groups, but differences did not reach statistical significance.

Prevalence of vitamin A deficiency (inflammation-adjusted retinol < 0.7 μmol/L) was 7.9% in the groups combined (7.7% unadjusted for inflammation), while 5% were zinc deficient (<9.9 μmol/L) (Table 4). Inflammation-adjusted retinol was greater by 0.1–0.1 μmol/L in the CFS groups relative to the control (*p* = 0.007) (Table 4), although reductions in risk of vitamin A deficiency were not statistically significant. Serum zinc was also lower in some supplemented groups relative to control (*p* = 0.017). Specifically, serum zinc was 4% lower (β (95% CI): 0.96 (0.91, 1.00)) in Plumpy’doz relative to control. Supplementation was associated with an increased risk of zinc deficiency overall. When testing individual supplements relative to control, the increased prevalence of deficiency was significant only in the rice-lentil group (PR: 2.9, 95% CI: 1.2–7.1).

## 4. Discussion

In 18-month-old children following year-long participation in a complementary food supplementation trial in rural Bangladesh, daily fortified complementary foods improved iron and vitamin A status relative to control. Hb, ferritin, and retinol were higher, and inflammation trended lower in supplemented children. Of note, increases in Hb, serum ferritin, and retinol occurred despite the relatively low prevalence of deficiencies, although the decrease in vitamin A deficiency did not reach statistical significance. Zinc concentrations did not differ by group, and zinc deficiency was low at about 4% in the control group. In two of the supplemented groups, zinc deficiency trended somewhat higher in the supplemented vs. the control group, but a chance finding or baseline imbalances could not be ruled out. Micronutrient status improvements persisted after adjusting micronutrient indicators for inflammation; thus, reduced inflammation did not explain the benefits of the CFSs. Rather, improved micronutrient status and reduced inflammation were likely independent benefits of the CFS intervention. This was plausible, as we also adjusted for inflammation in the control group. Overall, at 18 months of age, deficiency of iron, vitamin A, and zinc was found to be low in this population. Non-significant reductions in deficiency might, in part, be related to this low prevalence that our sample size was unable to detect.

In contrast to this, we found a very high burden of subclinical infection in this population, with 68% with either of the two inflammatory biomarkers being elevated. There was a 13–15% suggested reduction in inflammation, with three CFSs and a 23% reduction with WSB++, which was significant. Recently, small-quantity lipid-based supplementation has been found to significantly reduce childhood mortality by 27% in a meta-analysis of 18 trials [34]. A reduction in child morbidity, as previously shown in our study [21], and in inflammation, as shown in this analysis, may likely be pathways to improved child survival with complementary food supplementation.

Improved micronutrient status in groups receiving CFSs confirms maternal-reported adherence [21] and supports the effectiveness of the supplement formulations. The CFSs differed somewhat in micronutrient content (Supplementary Table S1), which might have translated to treatment effect differences. Children who received Plumpy’doz, which had 10.8 mg iron/100 g compared to 7–9 mg/100 g in the other supplements, had the largest increase in ferritin and Hb and the largest reduction in risk of iron deficiency. In contrast, higher vitamin A content of WSB++ and Plumpy’doz and greater zinc in chickpea and rice-lentil did not translate to a greater impact on vitamin A or zinc status. The supplements may have contained different quantities of anti-nutrients, such as phytates, due to different plant-based ingredients [35] which could affect micronutrient bioavailability [36]. The form of iron used in WSB++ was sodium iron-EDTA, preferred in the presence of phytates with wheat in the blend, and the form used in the chickpea and rice-lentil products was ferrous fumarate, which has higher absorption. Additionally, an increased abundance of iron (especially a highly bioavailable form) or zinc may compromise the absorption of the other due to a shared cellular transporter [37,38]. Serum zinc was lowest in the Plumpy’doz and rice-lentil groups, the groups with the greatest increases in serum ferritin, consistent with a tradeoff. Still, limitations of serum zinc as an indicator of status [39,40] and the low levels of deficiency are reasons for cautious interpretation of those findings.

In this sample of 18-month-olds in rural Bangladesh, iron, vitamin A, and zinc deficiencies were relatively uncommon and tended not to affect the same children. This contrasted recent national survey data reporting the prevalence of anemia and iron, vitamin A, and zinc deficiencies among rural pre-school children to be 37%, 11%, 21%, and 45%, respectively [41]. Mothers in all study arms received eight child feeding counseling sessions, which included messages about feeding diverse and micronutrient-rich foods. Still, we reported previously that participating children had inadequately diverse diets [42]. Concurrent supplementation programs, such as national semiannual vitamin A supplementation and widespread social marketing of “Sprinkles” micronutrient powders by the NGO BRAC [43], could have contributed, though in dietary data from the same study, reported Sprinkles consumption was nearly absent. Recent national estimates of vitamin A supplementation coverage in children 6–59 mo is just over 60% [44]. Groundwater rich in iron may benefit iron status; most households reported detectable iron in their drinking water, and children’s iron status markers were greater and prevalence of deficiency lower with increasing water iron, consistent with previous reports of iron absorbed from drinking water in adult women [45]. Water zinc content in Bangladesh also varies geographically [46,47] but, to our knowledge, its contribution to zinc status has never been evaluated. Other analytical factors that may have contributed to considerably different estimates of zinc deficiency in this population and in the national data include the proneness of zinc to ambient contamination, use of different methodological approaches across labs, and a lack of tools for standardizing zinc assessments among laboratories, unlike for the other analytes [48]. Still, we used best practices, including using trace-mineral-free collection systems and certified standard reference material, in each run to optimize the external validity of zinc values.

The randomized study design enabled attributing group-wise differences following 12 months of supplementation to the interventions, a tremendous strength of this study. However, there were limitations to our analysis. Assessment of baseline status would have ensured initial comparability among groups with respect to micronutrient status, although other baseline characteristics were examined, and most were highly comparable, and serum collection in infants in this context was not logistically feasible. Micronutrient status assessments were also limited in that only four micronutrient markers were measured and only in a subset of participants. Further, the biomarkers measured are imperfect indicators of status, though measurement of both Hb and ferritin for iron status and concurrent determination of CRP and AGP, as was done here, is currently recommended practice [49].

In this rural Bangladesh setting, a low prevalence of micronutrient deficiencies suggests that existing conditions protect iron, vitamin A, and maybe zinc status in many, but not all, children. Against that backdrop, one year of fortified complementary food supplementation with any of four formulations improved children’s iron and inflammation status, failed to reduce anemia, and increased vitamin A concentrations that did not translate into a reduction in deficiency. The effects on zinc status were inconsistent. Given impacts on growth, reduction of morbidity with corresponding improvements in inflammatory markers, and improved micronutrient status, fortified complementary food supplementation might offer a variety of health benefits to children in similar settings.

## Figures and Tables

**Table 1:** Laboratory methods [25].

Analyte	Method	Source	Reliability
Ferritin	Automated chemiluminescent immunoassay	Immulite 2000, Siemens Diagnostics, Tarrytown, NY, USA	Inter-assay CV < 6.5%
Hemoglobin	Point-of-care spectrometry	301B hemoglobinometer, HemoCue AB, Angelholm, Sweden	
Retinol	Reverse-phase HPLC	Acquity UPLC, Waters Corporation, Milford, MA, USA	Calibrated per run against the National Institute of Standards and Technology Standard Reference Material (NIST SRM) 968e; inter-assay CV < 15%
Zinc	Graphite furnace atomic absorption spectroscopy	AAnalyst 800, Perkin Elmer, Waltham, MA, USA	Calibrated within each run against NIST SRM 1598 and with lyophilized human serum product (Seronorm Trace Elements Serum, SERO, Billingstad, Norway) as a control; inter-assay CV < 8%
C-reactive protein	Automated chemiluminescent immunoassay	Immulite 2000, Siemens Diagnostics, Tarrytown, NY, USA	Inter-assay CV < 6.5%
A-1 acid glycoprotein	Radial immunodiffusion	Kent Laboratories, Bellingham, WA, USA	Commercial quality control material (Liquicheck 590X, Bio-Rad Laboratories, Inc., Hercules, CA, USA); CV = 12%

**Table 2:** Child and household characteristics of micronutrient assessment participants at enrollment by assigned supplementation group in a randomized complementary food supplementation trial in Bangladesh.

Characteristic	Control	Plumpy’doz	Chickpea	Rice-Lentil	WSB++	*p*-Value ^1^
*n*	*162*	*168*	*174*	*153*	*171*	
Sex, female	86 (53.1)	72 (42.9)	87 (50.0)	80 (52.3)	79 (46.2)	0.133
Length, cm, mean (SD)	64.3 (2.3)	64.2 (2.5)	64.2 (2.6)	64.0 (2.6)	63.9 (2.8)	0.500
Stunted	32 (19.8)	43 (25.6)	49 (28.2)	39 (25.5)	62 (36.3)	0.007
Weight, kg, mean (SD)	6.8 (0.8)	6.8 (0.9)	6.8 (0.9)	6.7 (0.9)	6.8 (0.9)	0.700
Underweight	26 (16.0)	30 (17.9)	40 (23.0)	30 (19.6)	43 (25.1)	0.198
Wasted	11 (6.8)	10 (6.0)	10 (5.7)	5 (3.3)	7 (4.1)	0.609
Mother’s education, any	125 (77.2)	126 (75.0)	128 (73.6)	117 (76.5)	139 (81.3)	0.534
Food insecurity, severe	16 (9.9)	19 (11.3)	21 (12.1)	16 (10.5)	25 (14.6)	0.730
Household owns cattle	76 (46.9)	91 (54.2)	83 (47.7)	68 (44.4)	84 (49.1)	0.519
Household owns land	115 (71.0)	122 (72.6)	126 (72.4)	112 (73.2)	115 (67.3)	0.752
Household has electricity	46 (28.4)	54 (32.1)	44 (25.3)	39 (25.5)	56 (32.7)	0.796
Drinking water iron ^2^						0.276
None	66 (40.7)	88 (52.4)	64 (36.8)	56 (36.6)	69 (40.4)	
A little	46 (28.4)	35 (20.8)	44 (25.3)	36 (23.5)	39 (22.8)	
Medium-high	50 (30.9)	45 (26.8)	66 (37.9)	58 (37.9)	63 (36.8)	

^1^*p*-values from linear or logistic GEE regression with clustering by sector. For drinking water iron, *p*-value came from an ordinal logistic regression model with variance adjusted for clustering of values within sectors using the “vce (cluster …)” command. ^2^ Mother’s perceived quantity of iron in household drinking water source.

**Table 3:** Inflammation biomarkers, the prevalence of inflammation and modeled differences, and prevalence ratios by supplementation group at age 18 months following one year of participation in a complementary food supplementation trial in rural Bangladesh.

	Supplementation Group				
	Control	Plumpy’doz	Chickpea	Rice-Lentil	WSB++
CRP, mg/L					
GM (95% CI) ^1^	1.58 (1.22–2.05)	1.32 (1.01–1.74)	0.94 (0.74–1.19)	1.23 (0.93–1.62)	1.26 (0.95–1.67)
β (95% CI) ^2,3^	-	0.84 (0.58, 1.21)	0.59 (0.41, 0.86)	0.78 (0.53, 1.14)	0.77 (0.53, 1.12)
AGP, g/L					
GM (95% CI) ^1^	1.10 (1.05–1.16)	1.06 (1.01–1.12)	1.03 (0.99–1.08)	1.07 (1.01–1.13)	1.02 (0.97–1.08)
β (95% CI) ^2,4^	-	0.96 (0.89, 1.03)	0.94 (0.87, 1.01)	0.97 (0.90, 1.04)	0.91 (0.84, 0.98)
Any inflammation (CRP > 5 mg/L or AGP > 1 g/L)					
*n* (%) ^1^	98 (68.1)	94 (59.9)	94 (59.1)	81 (59.1)	79 (54.1)
PR (95% CI) ^5,6^	1.00	0.85 (0.71, 1.03)	0.87 (0.73, 1.04)	0.84 (0.69, 1.03)	0.72 (0.57, 0.89)
High inflammation (CRP > 5 mg/L and AGP > 1 g/L)					
*n* (%) ^1^	137 (18.4)	31 (21.5)	30 (19.1)	25 (15.7)	24 (17.5)
PR (95% CI) ^5,7^	1.00	0.90 (0.58, 1.39)	0.73 (0.46, 1.16)	0.84 (0.69, 1.03)	0.72 (0.57, 0.89)

^1^ Observed values. ^2^ β-Coefficients (difference) and confidence intervals were estimated with GEE linear regression models with referent group as the control adjusted for sex and baseline stunting status and for clustering of observations by sector. Continuous CRP and AGP were log-transformed prior to analysis, and coefficients were back-transformed to the arithmetic scale. Coefficients for CRP and AGP were interpreted as 1-b_1_ percent difference compared to the control group. For example, CRP in the Plumpy’doz group was 1 − 0.84 = 16% lower than in the control group. ^3^ Overall *p*-value for complementary food groups as an indicator variable using GEE linear regression model = 0.09. ^4^ Overall *p*-value for supplementation groups as an indicator variable using the GEE linear regression model = 0.15. ^5^ Prevalence ratios and confidence intervals were estimated with GEE log-binomial regression with referent group as the control adjusted for sex and baseline stunting status and for clustering of observations by sector. ^6^ *p*-value for the set of supplementation group indicator variables in the GEE logistic regression model = 0.12. ^7^ *p*-value for the set of supplementation group indicator variables in the GEE logistic regression model = 0.73. Abbreviations: AGP, α-1 acid glycoprotein; CI, confidence interval; CRP, C-reactive protein; GEE, generalized estimating equation; GM, geometric mean; PR, prevalence ratio; WSB++, wheat soy blend plus plus.

**Table 4:** Micronutrient status indicators and modeled differences and prevalence ratios by supplementation group at age 18 months following one year of participation in a complementary food supplementation trial in rural Bangladesh.

	Supplementation Group				
	Control	Plumpy’doz	Chickpea	Rice-Lentil	WSB++
Ferritin, μg/L ^1^					
GM (95% CI) ^2^	32.8 (28.6–37.7)	47.0 (42.4–52.1)	38.3 (34.8–42.2)	44.5 (40.0–49.6)	38.1 (33.8–42.8)
β (95% CI) ^3,4^	-	1.5 (1.3, 1.8)	1.1 (1.0, 1.3)	1.3 (1.1, 1.5)	1.1 (1.0, 1.3)
Ferritin (<12 μg/L) ^1^					
*n* (%) ^2^	18 (12.5)	5 (3.2)	7 (4.4)	5 (3.6)	12 (8.2)
PR (95% CI) ^5,6^	1.0	0.2 (0.1, 0.6)	0.4 (0.2, 0.9)	0.4 (0.1, 0.9)	0.7 (0.4, 1.4)
Hemoglobin (g/L)					
Mean (95% CI) ^2^	118.0 (116.6–119.4)	121.7 (120.1–123.3)	119.4 (118.0–120.8)	119.3 (117.9–120.8)	119.9 (118.4–121.5)
β (95% CI) ^3,4^		4.0 (2.1, 5.9)	1.1 (−0.8, 3.0)	1.2 (−0.8, 3.2)	2.0 (0.1, 4.0)
Anemia (<110 g/L)					
*n* (%) ^2^	23 (15.8)	20 (12.6)	19 (11.9)	21 (15.1)	16 (10.8)
PR (95% CI) ^5,6^	1.0	0.7 (0.4, 1.2)	0.8 (0.5, 1.4)	1.0 (0.6, 1.7)	0.7 (0.4, 1.2)
Retinol (μmol/L) ^1^					
Mean (95% CI) ^2^	1.13 (1.07–1.19)	1.23 (1.17–1.29)	1.26 (1.19–1.32)	1.28 (1.21–1.35)	1.23 (1.17–1.30)
β (95% CI) ^3,4^	-	0.10 (0.02, 0.19)	0.13 (0.05, 0.21)	0.14 (0.06, 0.23)	0.12 (0.03, 0.20)
Retinol < 0.70 μmol/L ^1^					
*n* (%) ^2^	14 (10.1)	8 (5.4)	10 (6.7)	8 (6.0)	16 (11.3)
PR (95% CI) ^5,6^	1.0	0.5 (0.2, 1.2)	0.7 (0.3, 1.5)	0.6 (0.3, 1.4)	1.1 (0.6, 2.2)
Zinc (μmol/L)					
GM (95% CI) ^2^	12.9 (12.5–13.3)	12.3 (11.9–12.8)	13.2 (12.9–13.6)	12.5 (12.2–12.9)	12.9 (12.6–13.2)
β (95% CI) ^3,4^	-	0.96 (0.91, 1.00)	1.02 (0.98, 1.07)	0.97 (0.93, 1.02)	1.00 (0.95, 1.05)
Zinc < 9.9 μmol/L					
*n* (%) ^2^	5 (3.6)	10 (6.4)	4 (2.6)	13 (9.6)	4 (2.8)
PR (95% CI) ^5,6^	1.0	1.8 (0.7, 4.6)	0.7 (0.2, 2.4)	2.9 (1.2, 7.1)	0.9 (0.3, 2.7)

^1^ Ferritin and retinol values adjusted for inflammation status according to BRINDA methods [28,29]. ^2^ Observed values. ^3^ Coefficients and confidence intervals were estimated with GEE linear regression models with referent group being the control group adjusted for sex and baseline stunting status and for clustering of observations by sector. Iron status models were also adjusted for maternal-reported drinking water iron content. Ferritin and zinc were log-transformed prior to analysis, and coefficients were back-transformed to the arithmetic scale. For ferritin and zinc, coefficients were interpreted as 1-b1 percent difference compared to the control group. ^4^ Overall *p*-values for supplementation groups as an indicator variable using GEE linear regression models were <0.001 for ferritin; 0.001 for Hb; 0.01 for retinol, and 0.02 for zinc. ^5^ Prevalence ratios and confidence intervals were estimated with GEE log-binomial regression with referent group being the control adjusted for sex, baseline stunting status, and for clustering of observations by sector. Iron status models were also adjusted for maternal-reported drinking water iron content. ^6^ Overall *p*-values for supplementation groups as an indicator variable using GEE logistic regression models were 0.01 for iron deficiency; 0.55 for anemia; 0.29 for vitamin A deficiency; 0.01 for zinc deficiency. Abbreviations: CI, confidence interval; GM, geometric mean; PR, prevalence ratio; WSB++, wheat soy blend plus plus.

## Supplementary Materials

The following are available online at https://www.mdpi.com/2072-6643/12/5/1452/s1, Figure S1: Micronutrient status indicators by inflammation status in 18-mo-old children completing participation in a randomized-controlled complementary food supplementation trial in rural Bangladesh, Figure S2: Ferritin and hemoglobin values in 18-mo-old children completing participation in a one-year complementary food supplementation trial in Bangladesh by level of maternal-reported household drinking water iron content, Table S1: Nutrient value (per 100 g), for CFSs by age groups, Table S2: Design effects of the cluster randomized trial for micronutrient and inflammation biomarkers, Table S3: Child and household characteristics of supplementation trial participants at enrollment by inclusion status in the micronutrient assessment, Table S4: Micronutrient status unadjusted for inflammation and modeled differences and odds ratios by supplementation group at age 18 months following one year of participation in a complementary food supplementation trial in rural Bangladesh.
