# Supplementary material for: Micronutrient and Inflammation Status Following One Year of Complementary Food Supplementation in 18-Month-Old Rural Bangladeshi Children: A Randomized Controlled Trial
Source: Nutrients. 2020 May 18;12(5):1452. doi: 10.3390/nu12051452 (PMC7284655; doi:10.3390/nu12051452)
Supplement: Supplementary file 1 [file nutrients-12-01452-s001.zip › Supplementary figure 1.pdf]

Supplementary Figure 1. Micronutrient status indicators by inflammation status in 18-mo-old children completing participation in a randomized-controlled complementary food supplementation trial in rural Bangladesh[1,2,3]

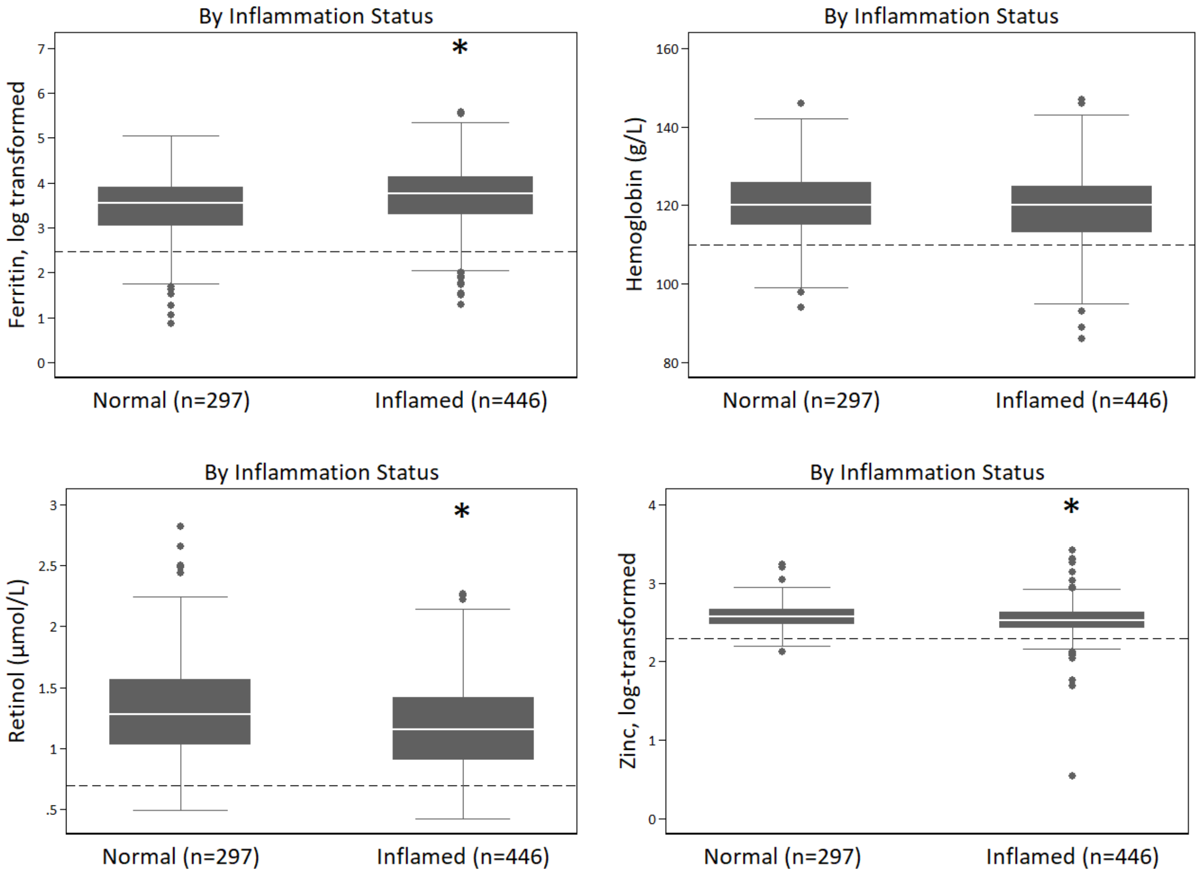

[1]Dashed lines indicate deficiency cutoffs for micronutrient markers

[2]Ferritin and zinc are displayed in log-transformed values and were log-transformed prior to GEE modeling

[3]Stars indicate a statistically significant difference ($p \leq 0.05$) in micronutrient marker between the inflamed and uninflamed groups based on GEE linear regression models adjusted for child sex and baseline stunting status and for clustering by sector.
