# Supplementary material for: Micronutrient and Inflammation Status Following One Year of Complementary Food Supplementation in 18-Month-Old Rural Bangladeshi Children: A Randomized Controlled Trial
Source: Nutrients. 2020 May 18;12(5):1452. doi: 10.3390/nu12051452 (PMC7284655; doi:10.3390/nu12051452)
Supplement: Supplementary file 1 [file nutrients-12-01452-s001.zip › Supplementary figure 2.pdf]

Supplementary Figure 2. Ferritin and hemoglobin values in 18-mo-old children completing participation in a one-year complementary food supplementation trial in Bangladesh by level of maternal-reported household drinking water iron content[1,2,3]

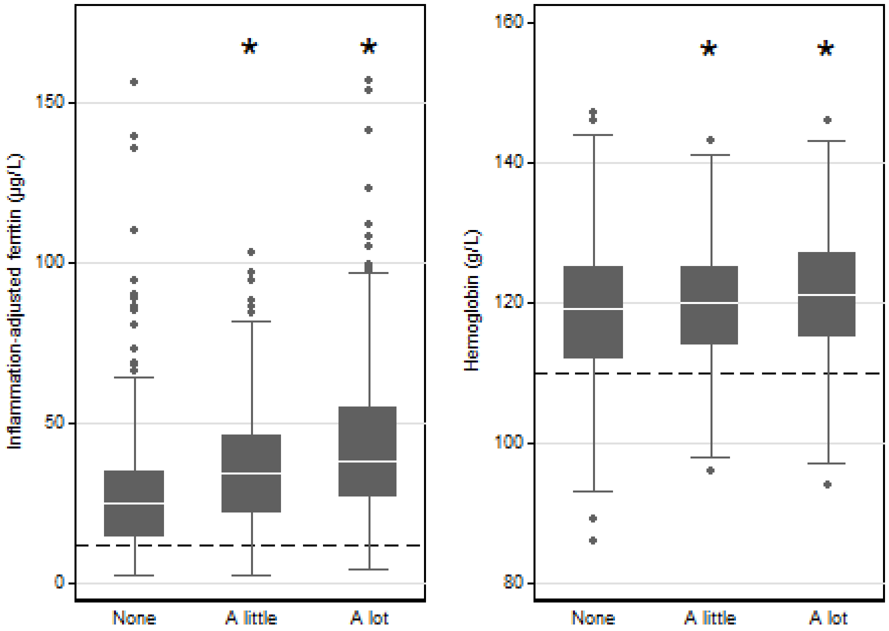

[1]Dashed lines indicate cutoffs for deficiency.
[2]Ferritin and retinol values adjusted for inflammation status according to BRINDA methods.[28,29] Ferritin was log-transformed prior to GEE modeling but is shown in the figure on the algorithmic scale.
[3]Stars indicate statistically significant difference ($p \leq 0.05$) relative to the "none" group (no iron reported in drinking water) based on GEE linear regression models adjusted for child sex and baseline stunting status and for clustering by sector.
