# Supplementary material for: Micronutrient and Inflammation Status Following One Year of Complementary Food Supplementation in 18-Month-Old Rural Bangladeshi Children: A Randomized Controlled Trial
Source: Nutrients. 2020 May 18;12(5):1452. doi: 10.3390/nu12051452 (PMC7284655; doi:10.3390/nu12051452)
Supplement: Supplementary file 1 [file nutrients-12-01452-s001.zip › Supplementary Table 1.docx]

Supplementary Table 1. Nutrient value (per 100 g), for CFSs by age groups (from Christian et al.^19^)

| **Nutrient** | **Content per 100 g of product** | | | |
| --- | --- | --- | --- | --- |
|  | **Plumpy'doz** | **Rice lentil** | **Chickpea** | **WSB++** |
| Amount given, g 6-11 mo | 23.2 | 25.7 | 23.6 | 32.7 |
| Amount given, g 12-18 mo | 46.4 | 51.4 | 47.2 | 63.4 |
| Kcal | 532 | 521 | 545 | 401 |
| Protein, g | 12.7 | 11 | 15 | 18 |
| Fat, g | 34 | 27 | 28 | 10 |
| Calcium, mg | 834 | 810 | 930 | 506 |
| Phosphorus, mg | 593 | 240 | 309 | 422 |
| Potassium, mg | 668 | 804 | 935 | 681 |
| Zinc, mg | 8.6 | 9.4 | 10.8 | 6.9 |
| Copper, mg | 0.65 | 0.8 | 1.1 | 0.3 |
| Iron^1^, mg | 19.4 | 13 | 15 | 8.1 |
| Iodine, mg | 119 | 131 | 143 | 59 |
| Selenium, μg | 37 | 29 | 32 | 24.7 |
| Magnesium, mg | 129 | 162 | 202 | 77 |
| Manganese, mg | 0.3 | 1.8 | 1.9 | 0.9 |
| Vitamin A, μg | 862 | 458 | 502 | 1039 |
| Vitamin D, μg | 0 | 22 | 24 | 12 |
| Vitamin E, mg | 12.9 | 19 | 20 | 14.7 |
| Vitamin K, μg | 0 | 44 | 48 | 47 |
| Thiamine, mg | 1.1 | 1.1 | 1.2 | 0.4 |
| Riboflavin, mg | 1.1 | 0.9 | 1 | 1.7 |
| Niacin, mg | 12 | 10 | 10 | 9 |
| Folic acid, μg | 345 | 389 | 501 | 181 |
| Vitamin B12, μg | 1.9 | 1.8 | 2 | 2.3 |
| Vitamin B6, mg | 1.1 | 1.1 | 1.2 | 1.1 |
| Vitamin C, mg | 65 | 31 | 34 | 92 |
| Pantothenic acid, mg | 4.3 | 4.3 | 4.2 | 2.2 |

^1^ Iron was in the form of ferrous fumarate in the chickpea and rice-lentil product and in the form of sodium iron-EDTA in WSB++. The form of iron in Plumpy’doz is not available.
