# Supplementary material for: Micronutrient and Inflammation Status Following One Year of Complementary Food Supplementation in 18-Month-Old Rural Bangladeshi Children: A Randomized Controlled Trial
Source: Nutrients. 2020 May 18;12(5):1452. doi: 10.3390/nu12051452 (PMC7284655; doi:10.3390/nu12051452)
Supplement: Supplementary file 1 [file nutrients-12-01452-s001.zip › Supplementary Table 2.docx]

Supplementary Table 2. Design effects of the cluster randomized trial for micronutrient and inflammation biomarkers

| Biomarker | Design Effect (%) |
| --- | --- |
| CRP | 1.066 |
| AGP | 1.068 |
| Ferritin | 1.329 |
| Hemoglobin | 1.005 |
| Retinol | 0.884 |
| Zinc | 1.158 |
