# Supplementary material for: Micronutrient and Inflammation Status Following One Year of Complementary Food Supplementation in 18-Month-Old Rural Bangladeshi Children: A Randomized Controlled Trial
Source: Nutrients. 2020 May 18;12(5):1452. doi: 10.3390/nu12051452 (PMC7284655; doi:10.3390/nu12051452)
Supplement: Supplementary file 1 [file nutrients-12-01452-s001.zip › Supplementary table 3.docx]

Supplementary Table 3. Child and household characteristics of supplementation trial participants at enrollment by inclusion status in the micronutrient assessment

| **Characteristic** | **Overall** | | |
| --- | --- | --- | --- |
|  | **Main study^1^** | **Sub-study** | **P-value^2^** |
|  | **n (%)** | **n (%)** |  |
| *n* | 4621 | 828 |  |
| Sex, female | 2324 (50.3) | 404 (48.8) | 0.437 |
| Length, cm, mean (SD) | 66.3 (1.6) | 64.1 (2.6) | 0.119 |
| Stunted | 1126 (24.4) | 225 (27.2) | 0.140 |
| Weight, kg, mean (SD) | 7.0 (0.4) | 6.8 (0.9) | 0.736 |
| Underweight | 916 (19.8) | 169 (20.4) | 0.796 |
| Wasted | 264 (5.7) | 43 (5.2) | 0.480 |
| Mother’s education, any | 3483 (75.4) | 635 (76.7) | 0.514 |
| Food insecurity, severe | 608 (13.2) | 97 (11.7) | 0.340 |
| Household owns cattle | 2364 (51.2) | 402 (48.6) | 0.204 |
| Household owns land | 3209 (69.4) | 590 (71.3) | 0.310 |
| Household has electricity | 1458 (31.6) | 239 (28.9) | 0.391 |
| Drinking water iron^3^ |  |  | 0.183 |
| None | 1730 (37.4) | 343 (41.4) |  |
| A little | 1191 (25.8) | 200 (24.2) |  |
| A lot | 1685 (36.5) | 282 (34.1) |  |

^1^Main study column includes participants in the parent trial but not the micronutrient status assessment sub-study.

^2^P-values from linear or logistic GEE regression with clustering by sector. For drinking water iron, p-value comes from an ordinal logistic regression model with variance adjusted for clustering of values within sectors using the “*vce(cluster …)*” command.

^3^Quantity of iron in the household drinking water source as perceived by the mother. Responses “a medium amount” and “a lot” were combined in the category labeled “a lot”.
