# Supplementary material for: Micronutrient and Inflammation Status Following One Year of Complementary Food Supplementation in 18-Month-Old Rural Bangladeshi Children: A Randomized Controlled Trial
Source: Nutrients. 2020 May 18;12(5):1452. doi: 10.3390/nu12051452 (PMC7284655; doi:10.3390/nu12051452)
Supplement: Supplementary file 1 [file nutrients-12-01452-s001.zip › Supplementary table 4.docx]

| Supplementary Table 4. Micronutrient status unadjusted for inflammation and modeled differences and odds ratios by supplementation group at age 18 months following one year of participation in a complementary food supplementation trial in rural Bangladesh | | | | | | |
| --- | --- | --- | --- | --- | --- | --- |
|  |  |  |  |  |  |  |
|  | **Overall** | **By supplementation group** | | | | |
|  |  | **Control** | **Plumpy'doz** | **Chickpea** | **Rice-Lentil** | **WSB++** |
| Ferritin, μg/L |  |  |  |  |  |  |
| GM (95% CI)^1^ | 36.8 (35.0-38.7) | 29.8 (26.0-34.2) | 43.7 (39.3-48.6) | 35.8 (32.6-39.3) | 41.3 (36.9-46.1) | 34.9 (31.0-39.1) |
| β (95% CI)^2^ |  | - | 1.55 (1.33, 1.81) | 1.16 (1.00, 1.35) | 1.33 (1.14, 1.56) | 1.15 (0.98, 1.34) |
| *P-value^3^* |  | *0.000* | | | | |
| Iron Deficient |  |  |  |  |  |  |
| n (%)^1^ | 50 (6.7) | 19 (13.2) | 6 (3.8) | 7 (4.4) | 6 (4.3) | 12 (8.2) |
| PR (95% CI)^4^ |  | 1.00 | 0.25 (0.10, 0.61) | 0.38 (0.17, 0.88) | 0.39 (0.16, 0.94) | 0.69 (0.35, 1.35) |
| *P-value^3^* |  | *0.009* | | | | |
| Retinol (μmol/L) |  |  |  |  |  |  |
| GM (95% CI)^1^ | 1.24 (1.21-1.27) | 1.15 (1.09-1.21) | 1.24 (1.18-1.30) | 1.27 (1.20-1.34) | 1.29 (1.22-1.36) | 1.25 (1.18-1.32) |
| β (95% CI)^2^ |  | - | 0.10 (0.01, 0.18) | 0.13 (0.04, 0.21) | 0.14 (0.05, 0.22) | 0.11 (0.03, 0.20) |
| *P-value^3^* |  | *0.015* | | | | |
| Vitamin A Deficient |  |  |  |  |  |  |
| n (%)^1^ | 55 (7.69) | 14 (10.07) | 10 (6.67) | 11 (7.33) | 7 (5.26) | 13 (9.09) |
| PR (95% CI)^4^ |  | 1.00 | 0.62 (0.30, 1.28) | 0.70 (0.34, 1.43) | 0.54 (0.24, 1.22) | 0.77 (0.38, 1.54) |
| *P-value^3^* |  | *0.574* | | | | |
| ^1^Observed values. | | | | | | |
| ^2^Coefficients and confidence intervals are estimated with GEE linear regression models with referent group control (CFC-only) adjusted for sex and baseline stunting status, and for clustering of observations by sector. Iron status models were also adjusted for maternal-reported drinking water iron content. Ferritin was log-transformed prior to analysis and coefficients are back-transformed to the arithmetic scale. Coefficients for ferritin are interpreted as 1-b_1_ percent difference compared to the control group. For example, ferritin in the Plumpy’doz group is 1-1.55= 55% higher than in the control group. | | | | | | |
| ^3^P-values are for the set of CFS group indicator variables in the GEE linear or logistic regression models. | | | | | | |
| ^4^Prevalence ratios and confidence intervals are estimated with GEE log-binomial regression with referent group CFC-only (control) adjusted for sex, baseline stunting status, and for clustering of observations by sector. Iron status models were also adjusted for maternal-reported drinking water iron content. | | | | | | |
| Abbreviations: CFS, complementary food supplement; CI, confidence interval; CP, chickpea CFS; GM, geometric mean; PD, Plumpy’doz; PR, prevalence ratio; RL, rice-lentil CFS; WSB, wheat soy blend *plus plus.* | | | | | | |
